# The anti-anxiety/depression effect of a combined complex of casein hydrolysate and γ-aminobutyric acid on C57BL/6 mice

**DOI:** 10.3389/fnut.2022.971853

**Published:** 2022-09-28

**Authors:** Lei Cai, Qian Tao, Wenzhi Li, Xiping Zhu, Chun Cui

**Affiliations:** ^1^School of Food Science and Engineering, South China University of Technology, Guangzhou, China; ^2^Infinitus (China) Co., Ltd., Guangzhou, China; ^3^College of Biological and Food Engineering, Anhui Polytechnic University, Wuhu, China; ^4^Guangdong Weiwei Biotechnology Co., Ltd., Guangzhou, China

**Keywords:** behavioral tests, casein hydrolysate, GABA, 5-HT, anxiety/depression

## Abstract

In view of a series of adverse side effects of drugs for anxiety/depression on the market at present, it is imminent to extract and develop novel anti-anxiety and depression drugs from plants and proteins (like casein hydrolysate) as adjuncts or substitutes for existing anti-anxiety and depression drugs. Consequently, this study investigated the improvement of the anxiety/depression function by the compound of casein hydrolysate and γ-aminobutyric acid (GABA) (casein hydrolysate: GABA = 4:1; CCHAA) on mice induced by chronic restraint stress-corticosterone injection. Animal experiments revealed that oral gavage administration of CCHAA significantly reversed the anxiety/depression-like behaviors. Compared to the model control group, body weights were increased after treatment with CCHAA groups [1.5, 0.75 mg/(g⋅d)]. As a diagnostic index of anxiety and depression, we assessed GABA and 5-HT levels in response to CCHAA ingestion. The GABA and 5-HT levels were increasingly enhanced by the CCHAA diet. In addition, histopathological changes in the hippocampus CA3 region of the anxious/depressed mice were also alleviated after the treatment with the CCHAA. Thus, the casein hydrolysate and GABA formula diets may induce beneficial effects on the mice with anxiety/depression.

## Introduction

Anxiety and depression are widely recognized as psychiatric disorders of global concern that impair human welfare (1). Features of anxiety disorders are generally thought to include cognitive, somatic, emotional, and behavioral changes (2–4). Anxiety disorders generally come in various forms, including high blood pressure, increased heart rate, sweating, fatigue, unpleasant feelings, nervousness, irritability, and restlessness (5–7). Further, in the absence of treatment, patients would gradually develop depression and sometimes even suicidal thoughts (8). Depression was the fourth largest disease burden in the world (8, 9), and with the development of society, it has shown an increasing trend of malignant development (10, 11). Depression is typically characterized by low mood, sadness or depression, and/or loss of interest or enjoyment in activities that were previously pleasurable (12, 13).

γ-aminobutyric acid (GABA) is not only an inhibitory neurotransmitter within the central nervous system, but also a key target for drug treatment of anxiety and depression (14, 15). Although the drugs currently on the market for anxiety/depression are therapeutically effective, they can cause a range of adverse side effects, including cognitive decline, and withdrawal symptoms (16). Therefore, the development of new anxiolytic and depressive drugs derived from plants and proteins provides a novel therapeutic option in order to find an adjunct or alternative to existing anxiolytic and depressive drugs (17, 18).

Casein is a protein rich in biologically active sequences, it can be obtained by enzymatically catalyzing the hydrolysis of proteins derived from microorganisms, animals, and plants, which can release these biologically active sequences under the action of specific endonucleases (such as trypsin, etc.) and similar physiological environmental conditions, so as to obtain casein bioactive peptides with biologically active (19, 20). Casein hydrolysis released a variety of active peptides, including ACE activity inhibitory peptides, immunomodulatory peptides, casein phosphopeptides that promote calcium absorption, peptides that promote the growth of Lactococcus, antithrombotic peptides, etc. (21, 22); and the hydrolysate also contains rich free amino acids, including abundant essential amino acids and oligopeptides containing 2–6 amino acid residues (23). Interestingly, Violle et al. showed that bovine αS1-casein trypsin hydrolysate exhibited anxiolytic-like activity in rats with the conditioned defensive burial and elevated plus maze models when injected intraperitoneally (24).

Meanwhile, there is growing support for the GABA hypothesis of depression as anxiety disorders and major depressive disorders are often found to have GABAergic deficits as a common pathophysiology, and thus GABA deficiency is a hallmark of anxiety disorders and major depressive disorder (25). However, as a highly complex psychiatric disorder, the pathogenesis of anxiety/depression remain obscure (26). The above single components played a role in the treatment of anxiety/depression through different mechanisms, but there is currently no research report on the combination of these components for the treatment of anxiety/depression. In addition, our research team used whey protein hydrolyzed peptides as raw materials for the treatment of anxiety/depression in the early stage and achieved good results (27, 28). Therefore, it is interesting to investigate whether compounds containing casein hydrolysate, whey protein hydrolyzed peptides and GABA, respectively, play a role in the treatment of anxiety/depression.

5-HT, also known as serotonin, is an important part of the biogenic aminergic neuroendocrine system (29, 30). As an important neurotransmitter in the rodent central nervous system, 5-HT is mainly involved in the regulation of various physiological activities such as cognitive function, emotional conditioning, appetite, sleep, and biological rhythms (31, 32). The 5-HT nervous system is divided into two independent nervous systems, the central 5-HT and the peripheral 5-HT, respectively (33). Peripheral 5-HT is mainly synthesized by tryptophan hydroxylase 1 (TPH1) and aromatic amino acid decarboxylase (AADC) in enterochromaffin cells, mast cells and 5-HTP cells, and stored in platelets (34). Central 5-HT is mainly synthesized by tryptophan hydroxylase 2 (TPH2) and aromatic AADC in 5-HT neurons and stored in presynaptic vesicles (35, 36). The synthesis amount of 5-HT in the center accounts for only about 5% of the total synthesis amount, and 95% of 5-HT is synthesized in the peripheral tissue of enterochromaffin cells as the main synthesis site (37). Studies have shown that both TPH1 and TPH2 dysfunction are closely related to anxiety-induced depression (38). Nevertheless, most drugs for the treatment of anxiety depression work by increasing 5-HT levels (39, 40).

This study aimed to investigate the improvement of the anxiety/depression function by CCHAA on mice induced by chronic restraint stress-corticosterone injection. The anxiety/depression-like behaviors, GABA and 5-HT synthesis, histopathological changes in the hippocampus CA3 region which is related to anxiety/depression in mice, were further studied.

## Materials and methods

### Materials and instruments

60 SPF grade C57BL/6 mice, 4 weeks old (21 ± 2 g) were obtained from Nanjing Junke Biological Engineering Co., Ltd. (Nangjing Jiangsu). The mice were adaptively reared for 7 days after purchase. During the experiment, the mice were fed in an environment of room temperature (25 ± 2)°C, relative humidity (50 ± 5)%, and a light/dark cycle of 12 h/12 h (every 5 cages; 320 mm × 215 mm × 170 mm), randomly provided with normal food and water with SPF level laboratory conditions. Moreover, humane care was given according to the 3R principles used in experimental animals. Corticosterone (batch number: 830F031) was purchased from Beijing Soleibo Technology Co., Ltd. (Beijing, China), and fluoxetine hydrochloride dispersible tablets (batch number: 9891A) were purchased from Eli Lilly Suzhou Pharmaceutical Co., Ltd. (Jiangsu, China). Casein hydrolysate (Lactium) was provided by Shanghai Tongyuan Food Technology Co., Ltd. GABA was provided by Nantong Licheng Biological Engineering Co., Ltd. (Nantong, China). Disposable filters, disposable syringes, 1.5 mL centrifuge tubes, 5 mL centrifuge tubes, pipettes, pipette tips, and beakers were purchased from Guangzhou Qianhui Instrument Equipment Co., Ltd. (Guangzhou Guangdong).

The animal experiments were approved by the Committee for the Care and Use of Laboratory Animals in the South China Agricultural University (IACUC No. 2018D047), and all experimental procedures were performed in compliance with the author Guidelines on Animal Ethics and Welfare for Veterinary Journals published by the International Association of Veterinary Editors for the protection of animals used for scientific purpose.

### Animal experiments

#### Establishment of animal model of anxiety/depression

**SCHEME 1:**
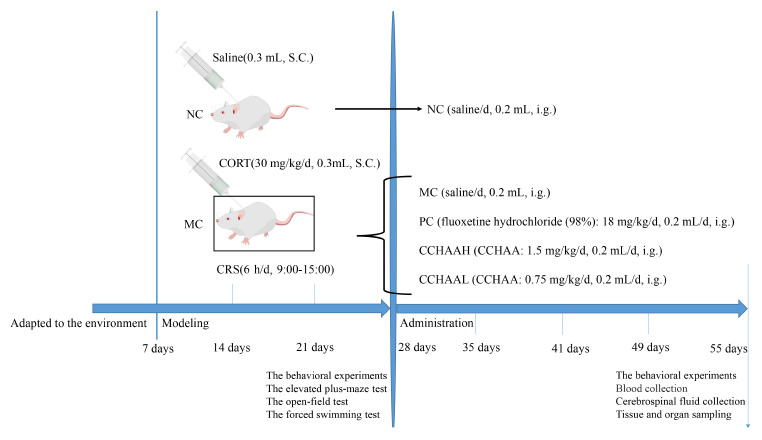
Diagram of the experimental paradigm. S.C., subcutaneous injection; i.g., intragastric administration.

The model experiment was carried out after mice adapted to the environment for 6 days. Mice were randomly assigned to 5 experimental groups (*n* = 12/group) and weighed weekly. 4 groups were subjected to chronic restraint stress (6 h/d, 9:00–15:00), and CORT (CORT dissolved in normal saline containing 0.1% dimethyl sulfoxide and 0.1% Tween-80) was injected subcutaneously in the volume of 0.02 mL/g body weight (30 mg/kg/d) (27, 28, 41). Normal control (NC) mice were fasted (6 h/d, 9:00–15:00) and given normal saline subcutaneously in the volume of 0.02 mL/g body weight (27, 28, 41). Finally, after 21 days of continuous modeling, the anxiety, and depression like behaviors of mice were tested. Incidentally, the mice with anxiety and depression were selected for gavage experiment (41). Further, these procedures were all shown in Scheme 1.

#### Animal different treatments

The normal control group (NC) was treatment with saline (0.2 mL/d), the positive control group (PC) was treatment with fluoxetine hydrochloride (98%) 18 mg/kg/d (0.2 mL/d) (28, 41), the model control group (MC) was treatment with saline (0.2 mL/d), the group was treatment with CCHAA at a high dose (CCHAAH) was 1.5 mg/(g⋅d) (0.2 mL/d), the group for treatment with CCHAA at a low dose (CCHAAL) was 0.75 mg/(g⋅d) (0.2 mL/d). To be explained: the CCHAA was administered to mice by oral gavage using a combination of a 12-gauge gavage needle and a 1.0 mL syringe. In detail: First, the mouse was immobilized so that its head, neck and body were in line. The specific method is as follows: the right hand pulls the mouse’s tail, the thumb, index finger, and middle finger of the left hand grab the mouse’s neck scalp, and the little finger and the nameless press the mouse’s tail. After grasping the mice, gavage can be performed. The specific method is as follows: the needle enters from the corner of the mouse’s mouth, presses the tongue, and pushes it inward carefully against the upper jaw. The gavage volume is usually 0.01–0.02 mL/g, and the maximum gavage volume of each mouse is no more than 0.8 mL. It should be noted that the immobilization of the mouse is the most important step in the gavage administration, and it needs to move quickly to reduce the discomfort of the animal.

#### The elevated plus-maze test

Behavioral experiments were performed on all mice during the day after 21 days of modeling (fourth week) and 4 weeks of treatment (eighth week) (42). The ZH-DSG elevated cross maze hardware and camera system used in this experiment were purchased from Anhui Zhenghua Instrument Equipment Co., Ltd. (Huaibei, Anhui, China). The maze consists of two open arms (30 cm long × 5 cm wide) and two closed arms (30 cm long × 5 cm wide × 15 cm deep) that extend from a common central platform (5 cm long × 5 cm wide) while the entire maze was raised to a height of 80 cm. In the whole test process, a calm and stable environment was ensured to obtain accurate results. Further, each mouse was placed separately in the center of the maze to face one of the open arms, and their behavior was recorded by video for 5 min. Then, the video was analyzed, and the number of entries (OE), and time (OT) of mice entering the open arm, the number of entries (CE) and time (CT) of mice entering the closed arm during the experimental period (5 min) were recorded, which was used to evaluate the anxiety like behavior of animals. It should be noted that between each test, the maze needs to be thoroughly cleaned with alcohol so that the animals will not be affected by the smell of the previous urine and feces (41, 43).

The equation for calculating the percentage of animal open arm entry times and the percentage of animal open arm residence time is as follows:


(1) $$\mathrm{OE}\%=\frac{\text{OE}}{\text{OE}+\text{CE}}\times 100\%$$

(2) $$\mathrm{OT}\%=\frac{\text{OT}}{\text{OT}+\text{CT}}\times 100\%$$

#### The open-field test

The ZH-ZFT open field experiment hardware and camera system used in this experiment was purchased from Anhui Zhenghua Instrument Equipment Co., Ltd. (Huaibei, Anhui, China). The experimental equipment is mainly composed of white PVC material, which is square box (40 cm long × 40 cm wide × 40 cm high). In addition, before each experiment or between two tests, the experimental equipment should be thoroughly cleaned with 70% ethanol aqueous solution and dried to eliminate the influence of urine and fecal odor left by the previous test. It should be noted that in order to obtain accurate results during the whole test process, it is necessary to ensure that the experiment is carried out in a quiet and stable environment. More importantly, the test also includes two stages: pre-test, in which the animal is gently placed in the top right corner of the box and allowed to adapt for 15 min. Further, a 10 min test was conducted 24 h after the end of the pre-test, in which each mouse was placed separately in the top right corner of the box again and allowed to move freely, and their movements and behaviors were also recorded *via* the digital camera system. After the experiment, the mice were immediately removed from the box and put back into the feeding cage. Finally, for video analysis, the analysis system is set to divide the box bottom into 16 equal sizes (10 cm × 10 cm) and record the time spent on each small square (28, 41). The percentage of time spent in the center was calculated skillfully by using the following formula to evaluate the anxiety like behavior of animals (42):


(3) Percentageofcentralresidencetime(%)

$$=\frac{\mathrm{Central}\,\mathrm{residence}\,\mathrm{time}}{\mathrm{Total}\,\mathrm{time}}\times 100\%$$

#### The forced swimming test

The ZH-QPT forced swimming hardware and camera system used in this experiment were purchased from Anhui Zhenghua Instrument Equipment Co., Ltd. (Huaibei, Anhui, China). All mice were subjected to forced swimming test during the day. In order to obtain accurate results, the experiment is carried out in a quiet and stable environment with the temperature is remained at 25 ± 1°C throughout the experiment. The test was conducted twice in 2 days. In the first stage, each mouse was gently placed into a glass cylinder (diameter 10 cm × 25 cm high) filled with water (water level height of 15 cm), so that each experimental mouse was adapted to swimming for 6 min. In the second stage test, 24 h after the end of adaptive swimming, the second stage experiment was started for a total of 6 min, in which the animals were placed in the cylinder and adapted to swimming for 1 min, and then a 5-min test was started. During the test, a digital camera system was used to track and record the movement and behavior of animals, and the total duration of immobility (in seconds) was measured (28, 41). More specifically, the standard of immobility in swimming was that when the experimental mice floated, they were observed to gently stroke or move with only one foot to keep their head above the water without struggling (44). And the mice were removed from the water and put back into the feeding cage immediately after the experiment.

#### Determination of biochemical indexes

After the behavioral experiments, mice were killed under the anesthetic effect of chloral hydrate. At the same time of removing the mouse eyeballs, the blood was immediately collected into the EDTA tube on ice, then centrifuged in a refrigerated centrifuge with the speed of 3,000 rpm/min and the temperature of 4°C for 15 min, and finally stored at –80°C until it was used. After that according to the manufacturer’s instructions (Shanghai QiaoDu Biotechnology Co., Ltd.), the contents of plasma corticotropin releasing hormone (CRH), adrenocorticotropic hormone (ACTH), and corticosterone (CORT) were determined by enzyme-linked immunosorbent assay (ELISA), and chemical colorimetry (45). Therefore, the prefrontal cortex, hypothalamus, and hippocampus were dissected accurately, weighed separately, and added with a certain amount of PBS to maintain their pH of 7.4, and snap frozen in liquid nitrogen for further use. Additionally, the specimen was thawed and still kept at a temperature of 4°C, and the supernatant was carefully collected and frozen for further use. Naturally, the double antibody sandwich method was used to determine mouse serotonin (5-HT), dopamine (DA), and γ- aminobutyric acid (GABA) levels. Firstly, the microplate was coated with purified mouse 5-HT, DA, and GABA antibodies to make solid-phase antibodies. Next, 5-HT, DA, and GABA were successively added to the micropores coated with monoclonal antibodies, and then combined with HRP labeled 5-HT antibodies to form antibody-antigen-enzyme labeled antibody complex. After thorough washing, the substrate TMB was added for color development. It should be reminded that TMB is converted into blue under the catalysis of HRP enzyme, and finally into yellow under the action of acid. In addition, the color depth was positively correlated with 5-HT, DA, and GABA in the samples. Finally, the absorbance (OD value) was measured by microplate reader at 450 nm wavelength, and the concentrations of 5-HT, DA, and GABA in mice were calculated by standard curve (41).

#### Histopathological examinations of hippocampus

The mice were deeply anesthetized with 4% chloral hydrate (0.1 mL/10 g intraperitoneally) and sacrificed by eyeball enucleation for blood sampling and spinal dislocation. Then, the hippocampus and hypothalamus were quickly removed on ice and rinsed with ice-cold saline solution. After absorbing water with absorbent paper, the hippocampus and hypothalamus were fixed with 4% paraformaldehyde fixative for 36 h. Next, the hippocampus and hypothalamus tissue were taken out from the fixative and placed in the dehydration box, and subsequently, the dehydration box was put into the dehydrator for dehydration with the following gradient alcohols: Subsequently, the dehydration box was put into the dehydrator for dehydration with the following gradient alcohols: 75% alcohol dehydration for 4 h, 85% alcohol dehydration for 2 h, 90% alcohol dehydration for 2 h, 95% alcohol dehydration for 1 h, and anhydrous ethanol for 1 h, dehydration of alcohol, and benzene for 8 min, dehydration of xylene for 10 min, melted paraffin wax I at 65°C for 1 h, melted paraffin wax II at 65°C for 1 h, and melted paraffin wax III at 65°C for 1 h. The wax-soaked hippocampal and hypothalamic tissues were embedded in an embedding machine and cooled at –20°C. After the wax solidified, the wax block was taken out of the embedding frame and placed in a paraffin microtome to slice with a thickness of 4 um. The sections were floated on a spreader in 40°C warm water to flatten the tissue, and then the tissue was picked up with a glass slide and baked in a 60°C oven. After the water was dried and the wax was baked, it was taken out and stored at room temperature for later use. Immediately afterward, the slices were placed in the following reagents in sequence: xylene I for 20 min, xylene II for 20 min, absolute ethanol I for 5 min, absolute ethanol II for 5 min, 75% alcohol for 5 min, and then washed with tap water. The tissue samples were stained with hematoxylin-eosin (H & E). The slices were placed in anhydrous ethanol I for 5 min, anhydrous ethanol II for 5 min, anhydrous ethanol II for 5 min, xylene I for 5 min, and xylene II for 5 min, and then sealed with transparent neutral gum. Finally, the samples were examined under an upright optical microscope (Nikon Eclipse E100, Nikon, Japan) and imaging system (Nikon DS-U3, Nikon, Japan) (41).

#### Data processing

One way ANOVA was performed with SPSS 17.0 (SPSS Inc., Chicago, IL, USA) statistical analysis software, and all tests were repeated three times. The analysis of the behavioral test was repeated 3 times because of errors, which need to be corrected by multiple experiments to ensure the universality and accuracy of the experiments. At the same time, it has the benefit of showing that the experiment can be repeated to show that the results are testable. Significance of differences between the means of the data in each group was assessed by using Duncan’s *post-hoc* test (*p* < 0.05). And the presentation of the data was in the form of mean ± variance. In all figures, statistically significant differences between group means were expressed generally as a, b, c, for *p* < 0.05, 0.01, and 0.001, respectively.

## Results and discussion

### Effects of CCHAA on body weight

During the whole experimental process, the body weight changes of the mice were shown in Table 1. In addition, the initial body weight of the mice in each group was 21 ± 2 g, and there was no significant difference (*p* > 0.05) among the groups. After 21 days of modeling, the body weight of the modeling mice in each group was significantly lower (*p* < 0.05) than that of the normal group, which indicated that our modeling was successful. Further, after 4 weeks of oral gavage administration, the body weights of mice in NC, PC, CCHAA (H and L) groups were significantly higher than (*p* < 0.05) in model group mice. In short, these results suggest that each of the tested samples exhibited efficacy in alleviating anxiety-like behaviors in anxiety-depressed mice after oral gavage administration for 4 weeks.

**TABLE 1:** Effects of several formulas on the body weight of mice.

Groups	Initial body weight (g)	Body weight at 21 days after modeling (g)	Body weight after 4 weeks of gavage (g)
NC	22.14 ± 1.28^a^	27.62 ± 1.37^a^	32.41 ± 2.12^a^
MC	21.00 ± 1.82^a^	25.00 ± 0.97^bc^	26.69 ± 1.22^b^
PC	21.78 ± 2.06^a^	26.10 ± 0.71^a^	31.57 ± 0.83^a^
CCHAAH	20.91 ± 1.44^a^	24.16 ± 1.49^bc^	29.32 ± 0.73^c^
CCHAAL	20.72 ± 0.90^a^	24.78 ± 1.91^bc^	28.44 ± 1.18^c^

Different letters in the same column of data indicate significant differences between groups (p < 0.05).

### The effects on the elevated plus-maze test

**FIGURE 1:**
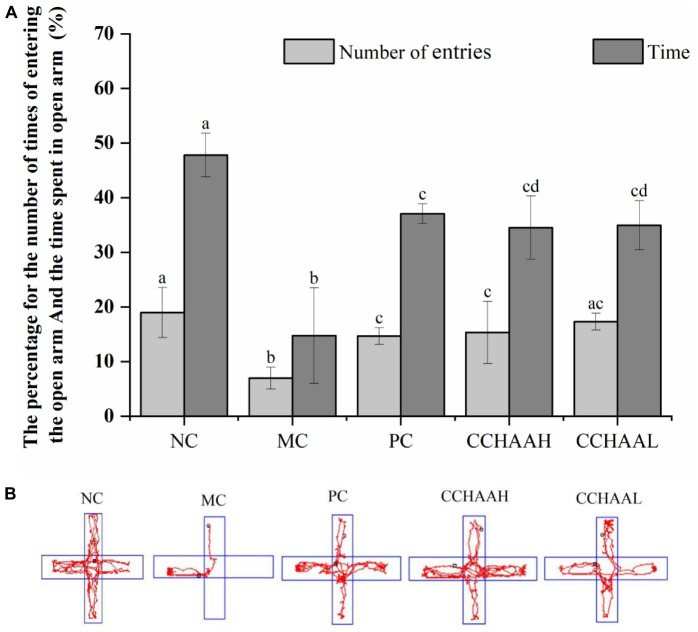
**(A)** The effects of CCHAA formula on the behaviors of mice in the elevated plus-maze test. Different letters indicate significant differences (*P* < 0.05). **(B)** The total movement tacked in EPM test: The red lines were the recorded motion tracks/routes of mice. The arms in the vertical direction were the closed arms, and those in the horizontal direction were the open arms.

Studies have shown that the elevated plus-maze is a method for rapidly screening mice for activity and anxiety-like behaviors without requiring pre-training of animals and the establishment of complex schedules (46). After 4 weeks oral gavage administration, compared with the mice in the model group, there were significant differences (*p* < 0.05) in the number of times of entering the open arms and the percentage of time spent in open arms in each test article-treated group, and the results are listed in Figure 1A. It can be known that the percentage of time spent in the open arm of the NC group was significantly (*p* < 0.05) higher than that of the other treatment groups, and the percentage of time spent in the closed arms was significantly lower (*p* < 0.05) than that of the other treatment groups. Obviously, the improvement effect of the number of times of entering the open arm was in the following order: NC > CCHAAL > CCHAAH > PC > MC; Meanwhile, the improvement effect of the percentage of residence time in the open arms was in the order: NC > PC > CCHAAL > CCHAAH > MC; And the percentage improvement effect of residence time in closed arms was in the following order: NC > PC > CCHAAH > CCHAAL > MC.

In the elevated plus-maze test, the arms in the horizontal direction are closed arms, the arms in the vertical direction are open arms, and the red lines are the recorded movement routes of the mice (Figure 1B). Movement loci and maximal depth into the open arms indicated that NC mice did not exhibit anxiety-like behaviors (Figure 1B) (47, 48). In the MC group, less movement in the closed arm and essentially no movement in the open arm were observed, and it was observed to stay in the corner of the closed arm, exhibiting pronounced anxiety-like behavior. According to the movement loci, the performance of the mice in each test article treatment group was comparable, but the maximum depth of entry into the open arms and the number of times the mice entered the open arms varied among these groups. These results show that each test article can alleviate the anxiety-like behavior of anxiety-depressed mice after 4 weeks of oral gavage administration.

### The effects on the open-field test

**FIGURE 2:**
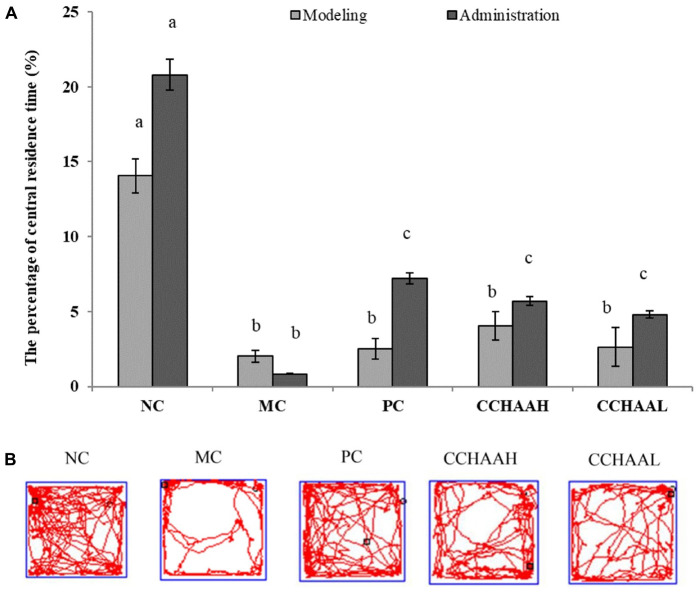
**(A)** The effects of CCHAA formula on the behaviors of mice in the open-field test. Different letters indicate significant differences (*P* < 0.05). **(B)** The total movement tracked in OFT: The black dots indicate where the mice started the test, with the black boxes showing where the mice stopped after the 10-min test, and the red lines were the recorded motion track/routes of mice.

The open field test is a simple and widely used method for evaluating activity and anxiety-like behavior in mice (46). The results after 4 weeks of gavage-treated mice with each test article are displayed in Figure 2A, where the percentage of central residence time of the mice in the NC group (20.80%) was significantly higher (*p* < 0.05) than those in the other test article-treated groups. The experimental results clearly demonstrated that the percentage of central residence time (0.84%) of the mice in the MC group was significantly lower (*p* < 0.05) than that of the mice in the other test groups. Furthermore, compared with MC mice, the percentage of central residence time in PC (7.19%) and CCHAAH (5.69%) was significantly increased (*p* < 0.05). The percent improvement effect of central residence time was as follows: NC > PC > CCHAAH >CCHAAL > MC.

After 4 weeks of drug oral gavage administration, the total movement loci (within 10 min) of each group are clearly depicted in Figure 2B: the black dot marks indicate where the mice started the test, the black square marks indicate where the mice stopped after 10 min of testing, and the red lines are the recorded movement loci or routes of the mice (28, 41). As a result, the movement loci of the mice in the MC group were mostly away from the center (around the edges and corners). In addition, compared with the mice in the MC group, the movement loci and the central area activity loci of the mice in treatment group were significantly (*p* < 0.05) increased. In conclusion, the fluoxetine hydrochloride and CCHAA have better effects in alleviating anxiety-like behaviors of mice in the open field test.

### The effects on the forced swimming test

**FIGURE 3:**
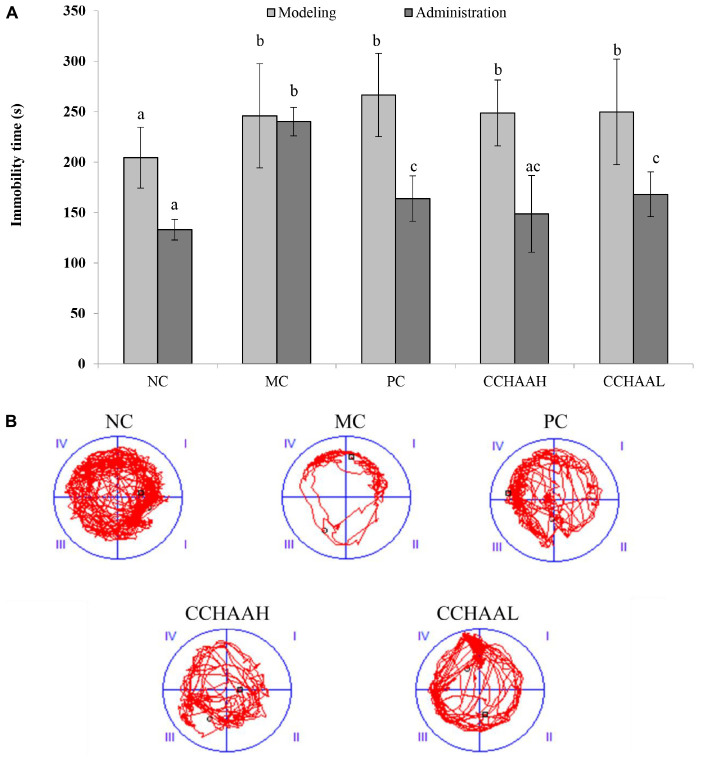
**(A)** The effects of CCHAA formula on the behaviors of mice in the forced swimming test. Different letters indicate significant differences (*P* < 0.05). **(B)** The total movement tracked in FST: The black dots indicate where the mice started the test, with the black boxes showing where the mice stopped after the 10-min test, and the red lines were the recorded motion track/routes of mice.

The forced swimming test as a stress model for depression is a typical method for evaluating antidepressant drugs and depression model systems, which is widely used in basic research on stress, psychiatry, and neuropharmacology (49). The results after 4 weeks of gavage treatment of each test article are exhibited in Figure 3A, and the results revealed that the mice in the MC group had the longest swimming immobility time (240.08 s), which was significantly higher (*p* < 0.05) than that of the PC and CCHAA groups. Nevertheless, the swimming immobility time in the NC group was the shortest (132.97 s). Although the swimming immobility time in the NC group was slightly lower than PC and CCHAA (H and L) groups, the difference was not statistically significant (*p* > 0.05). Besides that, it has been reported that the longer the swimming immobility time of experimental animals in the forced swimming test, the more severe the depression-like behavior (50).

The total movement traces in the FST are shown in Figure 3B. The MC mice mostly stayed around the edges with less movements, indicating obvious depressive-like behaviors (51). Whereas the mice of the PC and the CCHAA (H and L) groups exhibited significantly (*p* < 0.05) more movement including access to the center. Accordingly, the CCHAA formula can effectively eliminate the prolonged immobility time caused by CRS-CORT in mice, thereby effectively alleviating the depression-like behaviors in mice. Consequently, according to our results, both fluoxetine hydrochloride and CCHAA reduced the swimming immobility time in mice, which indicated that CCHAA improved behavior of depression-like in anxiety/depressed mice.

### The effects on the secretion of plasma hypothalamic-pituitary-adrenal axis

The dysfunction of hypothalamic-pituitary-adrenal (HPA) axis is currently recognized as one of the pathogeneses of anxiety and depression (52). Negative emotions such as anxiety and/or depression act as a stressor to stimulate the body to produce stress responses, which are felt in the cerebral cortex (53). After stimulation, the signal is transmitted to the hypothalamus, so that the hypothalamus releases corticotropin-releasing hormone (CRH); then, CRH stimulates the anterior pituitary to release ACTH; further, ACTH stimulates the adrenal gland to secrete CORT, which in turn has a negative feedback effect on CRH and ACTH, thereby affecting the functional state of the HPA axis (54). Studies have shown that plasma CRH and ACTH levels were significantly increased in patients with anxiety and depression (55). CORT, as the main plasma corticosteroid (75–90%), was essential for anxiety-depressive disorder and the body’s anti-stress response (including activation of tryptophan-2,3-dioxygenase, and immune system responses) (56, 57). Studies have also shown that chronic exposure to high levels of glucocorticoids reduced the expression of glucocorticoid receptors (presented in the hippocampus and provided negative feedback to the hypothalamus to prevent further release of glucocorticoids) (52, 58) and impaired the negative feedback regulation mechanism of the HPA axis (52, 59).

The result of the effect on plasma CRH and ACTH levels in anxiety-depressed mice are listed in Table 2. CRH (33.26 pg/mL) and ACTH (37.58 pg/mL) in NC mice were at normal levels, while The CRH and ACTH levels in MC group were significantly higher (*p* < 0.05) than those in the NC group. After 4 weeks oral gavage administration with CCHAA, the levels of plasma CRH and ACTH in the mice were significantly lower (*p* < 0.05) than those in MC group. According to the previous studies (27, 28, 41), CCHAA have the effect on improving the levels of plasma CRH and ACTH in anxiety/depression mice.

**TABLE 2:** The effects on the secretion of plasma hypothalamic-pituitary-adrenal (HPA) axis (*n* = 12).

Groups	CRH (pg/mL)	ACTH (pg/mL)
NC	33.26 ± 1.85^a^	37.58 ± 2.90^a^
MC	60.29 ± 6.52^b^	91.82 ± 10.60^b^
PC	45.85 ± 1.18^c^	59.84 ± 5.92^c^
CCHAAH	41.70 ± 2.02^c^	57.46 ± 5.31^c^
CCHAAL	42.56 ± 2.84^c^	64.31 ± 1.78^c^

Different letters in the same column of data indicate significant differences between groups (p < 0.05).

### The effects on neurotransmitter secretion in the brain tissue

After the 4-week treatment of this study, the NC mice had normal 5-HT, DA, and GABA level in the brain tissue (Table 3). The level of 5-HT, DA and GABA in the MC group were significantly lower (*p* < 0.05) than those in the NC group, which indicated that chronic restraint stress combined with subcutaneous injection of CORT on the back could induce a significant decrease (*p* < 0.05) in the levels of 5-HT, DA, and GABA in the brain tissue. The levels of 5-HT and GABA in the brain tissue of PC group (fluoxetine hydrochloride treatment group) and CCHAA (casein hydrolysate: GABA) mice were significantly higher than (*p* < 0.05) in the MC group. Compared with mice in MC group, DA level in brain tissue of PC and CCHAAH-treated mice were significantly increased (*p* < 0.05). There was no significant different (*p* > 0.05) between MC and CCHAAL group, and CCHAAL was slightly higher than MC mice.

**TABLE 3:** The effects on neurotransmitter secretion in the brain tissue (*n* = 12).

Groups	5-HT (ng/mL)	DA (ng/mL)	GABA (μmol/L)
NC	391.53 ± 55.30^a^	7.03 ± 0.51^ac^	7.39 ± 0.41^a^
MC	285.79 ± 26.00^b^	5.43 ± 1.32^b^	4.54 ± 1.10^b^
PC	360.43 ± 14.29^ac^	8.11 ± 0.23^c^	6.81 ± 1.28^a^
CCHAAH	325.50 ± 18.67^c^	8.57 ± 0.63^c^	7.19 ± 1.66^a^
CCHAAL	332.68 ± 76.28^c^	6.40 ± 1.20^b^	7.19 ± 1.13^a^

Different letters in the same column of data indicate significant differences between groups (p < 0.05).

### The effects on the histopathology of hippocampal CA3 region

**FIGURE 4:**
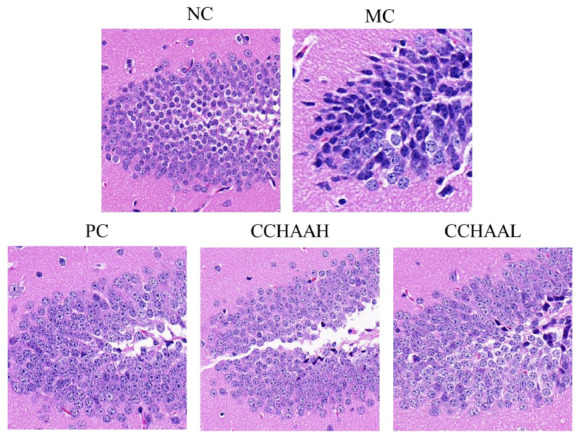
The histopathological alterations in the hippocampal CA3 regions caused by CCHAA.

The hippocampus is a high-level regulatory center of the subcutaneous center of emotional management (59). Long-term CORT exposure will atrophy and reduce the number of vertebral cells in the hippocampal CA3 region of patients with anxiety and depression, resulting in structural and functional damage to the hippocampus (60–62). Hence, the histopathological observation of the mouse hippocampus was performed, and the results of H&E staining of the hippocampal CA3 region were illustrated in Figure 4. It can be seen that the hippocampal structure of the NC mice was clear, the pyramidal cells were closely arranged, and the tissue cell shape (in oval shape) was regular. Compared with the NC mice, the number of pyramidal cells in CA3 area of MC mice was significantly reduced, cytoplasmic vacuolation, nuclear pyknosis, hyperchromatic cytoplasm, cell membrane shrinkage, irregular cell morphology, and blurred cell boundaries. After 4-week of treatment with fluoxetine hydrochloride and CCHAA, compared with the MC group, the number of cells in the CA3 region of hippocampus was significantly increased (*p* < 0.05), neatly arranged, the cell morphology was basically normal, and the outline of the hippocampus was clear. Despite the presence of pyramidal cell lesions in the CA3 area, the histopathological sections of the hippocampal CA3 region of CCHAA-treated mice were more similar to those of NC mice. In conclusion, fluoxetine hydrochloride and CCHAA have the effect on improving the pathological changes in the CA3 region of hippocampus in mice with anxiety/depression.

Here, we have shown that CCHAA formulation has an improving effect on anxiety/depressive function in mice. Specifically, the effects of CCHAA formula on anxiety-depression-like behaviors including weight change, elevated plus maze test, open-field test, and forced swimming test in anxiety/depressed mice, the effects on plasma HPA axis secretion, the effects on neurotransmitter secretion in brain tissue, and the effects on histopathology in hippocampal CA3 region, were studied, respectively. Fluoxetine, a widely used new antidepressant drug (63), was used as a positive control in the experiment. It can be found from these experimental results that the CCHAA (H and L) formulations and the PC groups effectively reduced anxiety/depression-like behaviors in mice subjected to chronic stress. In addition, the effects of CCHAA (H and L) formula groups were very similar to that of PC group, which indicates that CCHAA (H and L) formulas could achieve the same degree of anti-anxiety/depression effect as fluoxetine.

It was found in our previous work that Trp oligopeptides (EW and WPH) could improve anxiety/depression function and four possible mechanisms by which Trp oligopeptides enhanced 5-HT synthesis and anti-anxiety/depression effects were proposed (27, 28). Accordingly, we can fully guess that the possible mechanism of Trp oligopeptide enhancing 5-HT synthesis and anti-anxiety/depression effects may be very similar to that of the CCHAA (H and L) formulations (27).

## Conclusion

This study aimed to investigate the improvement of the anxiety/depression function by the compound of casein hydrolysate and γ-aminobutyric acid (GABA) (casein hydrolysate: GABA = 4:1; CCHAA) on mice induced by chronic restraint stress-corticosterone injection. The anxiety/depression-like behaviors, GABA and 5-HT synthesis, histopathological changes in the hippocampus CA3 region which is related to anxiety/depression in mice, was further studied. Animal experiments revealed that oral gavage administration of CCHAA significantly reversed the anxiety/depression-like behaviors. Compared to the model control group, body weights were increased after treatment with CCHAA groups [1.5, 0.75 mg/(g⋅d)]. As a diagnostic index of anxiety and depression, we assessed GABA and 5-HT levels in response to CCHAA ingestion. The GABA and 5-HT levels were increasingly enhanced by the CCHAA diet. In addition, histopathological changes in the hippocampus CA3 region of the anxious/depressed mice were also alleviated after the treatment with the CCHAA. Thus, the casein hydrolysate and GABA formula diets may induce beneficial effects on the mice with anxiety/depression. According to the demonstrated effects of the CCHAA formula in reversing the anxiety/depression-like behaviors, regulating the HPA axis, alleviating the histopathological changes in the hippocampus CA3 region of the anxious/depressed mice, the CCHAA formula might be an effective therapeutic product to help treat anxiety/depression. Moreover, it is particularly important that it also provides a new direction and guiding significance for our team to further development of new drugs for the treatment of anxiety/depression.

## Data availability statement

The original contributions presented in this study are included in the article/supplementary material, further inquiries can be directed to the corresponding author/s.

## Ethics statement

The animal study was reviewed and approved by the Committee for the Care and Use of Laboratory Animals in the South China Agricultural University [IACUC No. 2018D047].

## Author contributions

LC, CC, and WL conceived the idea. LC and QT conducted the statistical analyses. LC and XZ drafted the manuscript. XZ and QT carried out experiments. All authors contributed to manuscript revision, read, and approved the submitted version.
